# Compound C inhibits nonsense-mediated RNA decay independently of AMPK

**DOI:** 10.1371/journal.pone.0204978

**Published:** 2018-10-05

**Authors:** Abigael Cheruiyot, Shan Li, Andrew Nickless, Robyn Roth, James A. J. Fitzpatrick, Zhongsheng You

**Affiliations:** 1 Department of Cell Biology & Physiology, Washington University School of Medicine, St. Louis, Missouri, United States of America; 2 Department of Neuroscience, Washington University School of Medicine, St. Louis, Missouri, United States of America; 3 Department of Biomedical Engineering Washington University, St. Louis, Missouri, United States of America; 4 Center for Cellular Imaging, Washington University School of Medicine, St. Louis, Missouri, United States of America; Korea University, REPUBLIC OF KOREA

## Abstract

The nonsense mediated RNA decay (NMD) pathway safeguards the integrity of the transcriptome by targeting mRNAs with premature translation termination codons (PTCs) for degradation. It also regulates gene expression by degrading a large number of non-mutant RNAs (including mRNAs and noncoding RNAs) that bear NMD-inducing features. Consequently, NMD has been shown to influence development, cellular response to stress, and clinical outcome of many genetic diseases. Small molecules that can modulate NMD activity provide critical tools for understanding the mechanism and physiological functions of NMD, and they also offer potential means for treating certain genetic diseases and cancer. Therefore, there is an intense interest in identifying small-molecule NMD inhibitors or enhancers. It was previously reported that both inhibition of NMD and treatment with the AMPK-selective inhibitor Compound C (CC) induce autophagy in human cells, raising the possibility that CC may be capable of inhibiting NMD. Here we show that CC indeed has a NMD-inhibitory activity. Inhibition of NMD by CC is, however, independent of AMPK activity. As a competitive ATP analog, CC does not affect the kinase activity of SMG1, an essential NMD factor and the only known kinase in the NMD pathway. However, CC treatment down-regulates the protein levels of several NMD factors. The induction of autophagy by CC treatment is independent of ATF4, a NMD target that has been shown to promote autophagy in response to NMD inhibition. Our results reveal a new activity of CC as a NMD inhibitor, which has implications for its use in basic research and drug development.

## Introduction

First discovered in *S*. *cerevisiae*, nonsense mediated mRNA decay (NMD) is an evolutionarily conserved RNA quality control pathway that targets aberrant RNAs with PTCs for degradation[1]. Translation of PTC-containing mRNAs generates aberrant protein products, which may have pathological effects on the cell. Therefore, NMD plays an important protective role in the cell. It is believed that NMD modulates the clinical outcome of approximate 1/3 of human genetic disorders and many forms of cancer caused by mutations that lead to nonsense mRNAs[2]. In addition to downregulating mutant transcripts, more recent studies indicate that NMD also regulates the expression of ~10% of mRNAs bearing NMD-inducing features (e.g. PTCs caused by alternative splicing or programmed intron retention, upstream open reading frames (uORFs), intron-containing 3’ UTRs, exceedingly long 3’ UTRs), as well as many noncoding RNAs[3, 4]. Consequently, NMD as well as its dynamic regulation play an important role in many physiological processes, such as embryonic development, neurogenesis, myogenesis and stress responses[3, 5–9].

Small-molecule inhibitors or enhancers of NMD offer critical tools for dissecting the NMD pathway and its physiological functions. They also have the potential for treating certain genetic disorders and cancer[2, 10]. While active NMD renders many dominant mutations recessive by degrading transcripts encoding abnormal proteins with dominant activities, it can exacerbate the phenotypes of many disorders by preventing the synthesis of truncated protein products with normal function[10]. Therefore, inhibition of NMD is an attractive therapeutic approach for treating certain diseases where the protein products of the corresponding nonsense mRNAs are fully or partially functional. Furthermore, NMD inhibitors can be combined with drugs that promote translation readthrough at PTCs (e.g. PTC124 and aminoglycosides such as G418 and gentamicin) to produce full-length protein products[11–13]. As a proof of concept, co-treatment with NMD inhibitor NMDI14 and G418 restored full-length p53, and subsequent cell death in cells expressing nonsense p53 mRNA[14]. Similar effects have been observed for siRNA-mediated depletion of UPF1 and gentamicin in promoting the production of full length CFTR protein in cell lines derived from cystic fibrosis patients that carry nonsense mutations in the gene[15]. Inhibition of NMD also has the potential to improve cancer immunotherapy[16]. In cancer cells, nonsense mRNAs produced by aberrant splicing or frame-shift mutations encode proteins with novel epitopes, therefore, NMD inhibition is expected to increase the levels of cancer antigens for immune detection[16]. Several NMD inhibitors with different mechanisms of action have been previously identified. NMDI-1 disrupts the interaction of the NMD factors UPF1 and SMG5, whereas NMDI-14 disrupts the interaction of UPF1 with SMG7[14, 17]. Pateamine A inhibits the function of the EJC factor eIF4III in NMD[18]. Cardiac glycosides (e.g. ouabain, digoxin, digitoxin, lanatoside C and proscillaridin) and 5-azacytidine have been shown to inhibit NMD indirectly by increasing intracellular calcium levels and by inducing MYC expression, respectively[19, 20].

In this study, we have identified compound C (6-[4-(2-Piperidin-1-ylethoxy) phenyl]-3-pyridin-4-ylpyrazolo [1,5-a] pyrimidine) as a new cell-permeable, small-molecule inhibitor of NMD in human cells. Compound C (CC) was first identified as an inhibitor of the metabolic sensor kinase AMPK in a chemical screen[21]. CC inhibits the kinase activity of AMPK by competing with ATP for binding, with Ki of 109 ± 16 nM in the absence of AMP[21]. The binding of CC to AMPK also prevents its activation by AICAR or metformin, the most prescribed drug for type II diabetes[21]. Interestingly, CC was also identified in a functional screen in zebrafish as a compound (Dorsomorphin) that perturbs dorsoventral axis formation[22]. CC exerts this activity by inhibiting bone morphogenetic protein (BMP) type 1 receptors ALK2, ALK3, and ALK6, preventing BMP-mediated SMAD 1/5/8 phosphorylation, target gene transcription and osteogenic differentiation[22]. In addition to AMPK and ALKs, CC was found to inhibit several other kinases, including ERK8, MNK1, PHK, MELK, DYRK isoforms, HIPK2, Src, and Lck, with similar or even greater potency[23]. Thus, CC is a selective, but not specific, inhibitor of AMPK, although it is widely used as a tool for AMPK studies[24–29]. A recent study shows that CC can induce autophagy, which would be contrary to the finding that activation of AMPK, but not its inhibition, promotes autophagy[30–32]. However, the ability of CC to induce autophagy is AMPK-independent[30]. Interestingly, disruption of NMD activity also results in activation of autophagy, which is, in part, due to the stabilization of the transcripts of ATF4, a transcription factor that promotes expression of multiple autophagy genes including LC3B and ATG5[33]. These observations prompted us to test the possibility that CC inhibits NMD, which in turn leads to autophagy induction. By examining the effects of CC on a highly effective NMD reporter as well as endogenous NMD target transcripts, we demonstrate that CC indeed possesses a previously unrecognized activity in inhibiting NMD in human cells. The ability of CC to suppress NMD is not mediated through the inhibition of AMPK or SMG1, the only known kinase in the NMD pathway, but it down-regulates protein levels of several core NMD factors. Although CC treatment causes upregulation and stabilization of the NMD target ATF4, this effect is apparently not responsible for the induction of autophagy by CC.

## Results

### CC inhibits NMD activity in human cells

**Fig 1:**
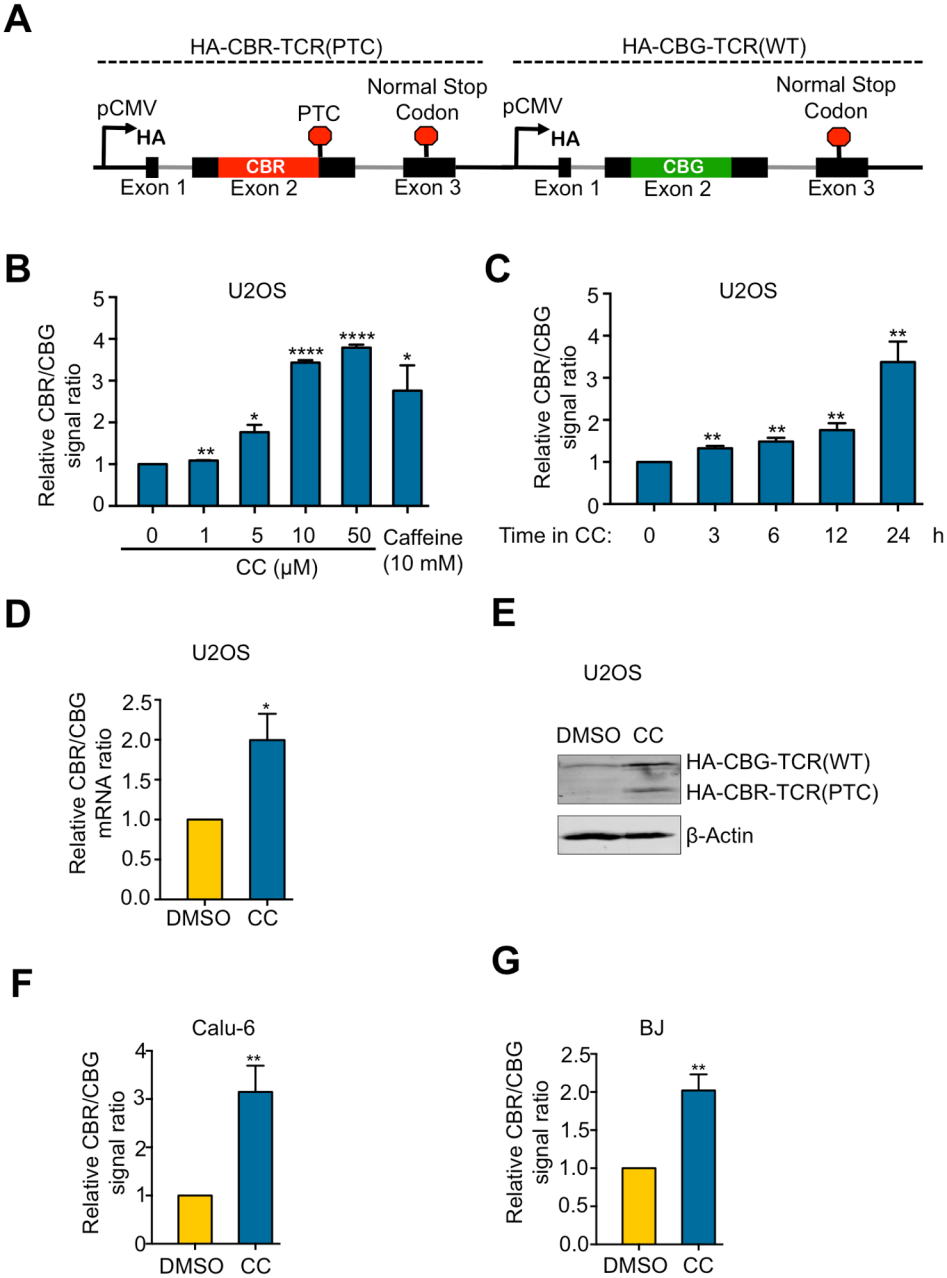
CC inhibits NMD in human cells. A. Schematic diagram of the dual color bioluminescence-based NMD reporter construct containing CBR-TCRβ(PTC) and CBG-TCRβ(WT) transcription units. B. Ratios of CBR to CBG bioluminescence signals in U2OS cells stably expressing a dual color bioluminescence-based NMD reporter (hereafter referred to as U2OS reporter cells). Cells were treated with indicated concentrations of CC, or caffeine for 24 hours before imaging. The CBR/CBG ratio of the DMSO alone control was normalized to 1. Data represent the mean ± SD of three independent experiments. ****p ≤ 0.0001; **p ≤ 0.01; *p ≤ 0.05 (paired t-test). C. Ratios of CBR to CBG bioluminescence signals in U2OS reporter cells treated with DMSO or CC (10 μM) for the indicated times. The CBR/CBG ratio of the 0-hour time point was normalized to 1. Data represent the mean ± SD of three independent experiments. **p ≤ 0.01 (paired t-test). D. Ratios of CBR to CBG reporter mRNAs in U2OS reporter cells treated with DMSO or CC (10 μM) for 24 hours. The CBR/CBG mRNA ratio of the DMSO alone control was normalized to 1. Data represent the mean ± SD of three independent experiments. *p ≤ 0.05 (paired t-test). E. Western blot result of the NMD reporter proteins (HA-tagged) after 24-hour treatment of U2OS reporter cells with DMSO or CC (10 μM). F. Ratios of CBR to CBG bioluminescence signals in Calu-6 cells infected with adenoviruses expressing the NMD reporter after 24-hour treatment with DMSO or CC (10 μM). The CBR/CBG ratio of the DMSO alone control was normalized to 1. Data represent the mean ± SD of three independent experiments. **p ≤ 0.01 (paired t-test). G. Ratios of CBR to CBG bioluminescence signals in BJ cells infected with adenoviruses expressing the NMD reporter after 24-hour treatment with DMSO or CC (10 μM). The CBR/CBG ratio of the DMSO alone control was normalized to 1. Data represent the mean ± SD of three independent experiments. **p ≤ 0.01 (paired t-test).

To determine whether CC can modulate NMD efficiency in human cells, we used a previously-developed bioluminescence-based reporter system, which can accurately measure NMD activity in mammalian cells using multiple assays[19]. This reporter contains two transcription units that express a PTC-containing TCRβ minigene fused to CBR luciferase (CBR-TCRβ(PTC)) and a wild type TCRβ minigene fused to CBG luciferase (CBG-TCRβ(WT)), respectively (Fig 1A). NMD activity is measured as the ratio of CBR-TCRβ(PTC) to CBG-TCRβ(WT) at the levels of mRNA, protein, or luciferase activity, with an increase in the ratio representing NMD inhibition[19]. To determine the effects of CC on NMD, we treated human U2 osteosarcoma (U2OS) cells stably expressing the reporter with CC and then measured NMD activity by bioluminescence imaging. CC inhibited NMD in a dose- and time-dependent manner, with ~ 3-fold inhibition of NMD of the reporter observed after 24-hr treatment with CC at 10 μM, a concentration commonly used to inhibit AMPK *in vivo* and *in vitro* (Fig 1B and 1C). This level of inhibition is similar to that caused by treatment with caffeine (10 mM, 24 hrs), an inhibitor of SMG1 (Fig 1B)[17], or by shRNA-mediated knockdown of NMD factors such as SMG1, UPF1 and UPF2[19].

To confirm the results obtained from bioluminescence imaging, we measured CBR and CBG mRNA and protein levels using RT-qPCR and western blot, respectively. Consistent with the results of bioluminescence imaging, CC treatment increased the ratio of CBR-TCRβ(PTC) to CBG-TCRβ(WT) at both mRNA and protein levels (Fig 1D and 1E). Treating the human lung cancer cell line Calu-6 or non-transformed BJ human fibroblasts with CC also resulted in NMD inhibition as measured by the NMD reporter (Fig 1F and 1G), indicating that the effect of CC on NMD is not a cell line-specific phenomenon.

**Fig 2:**
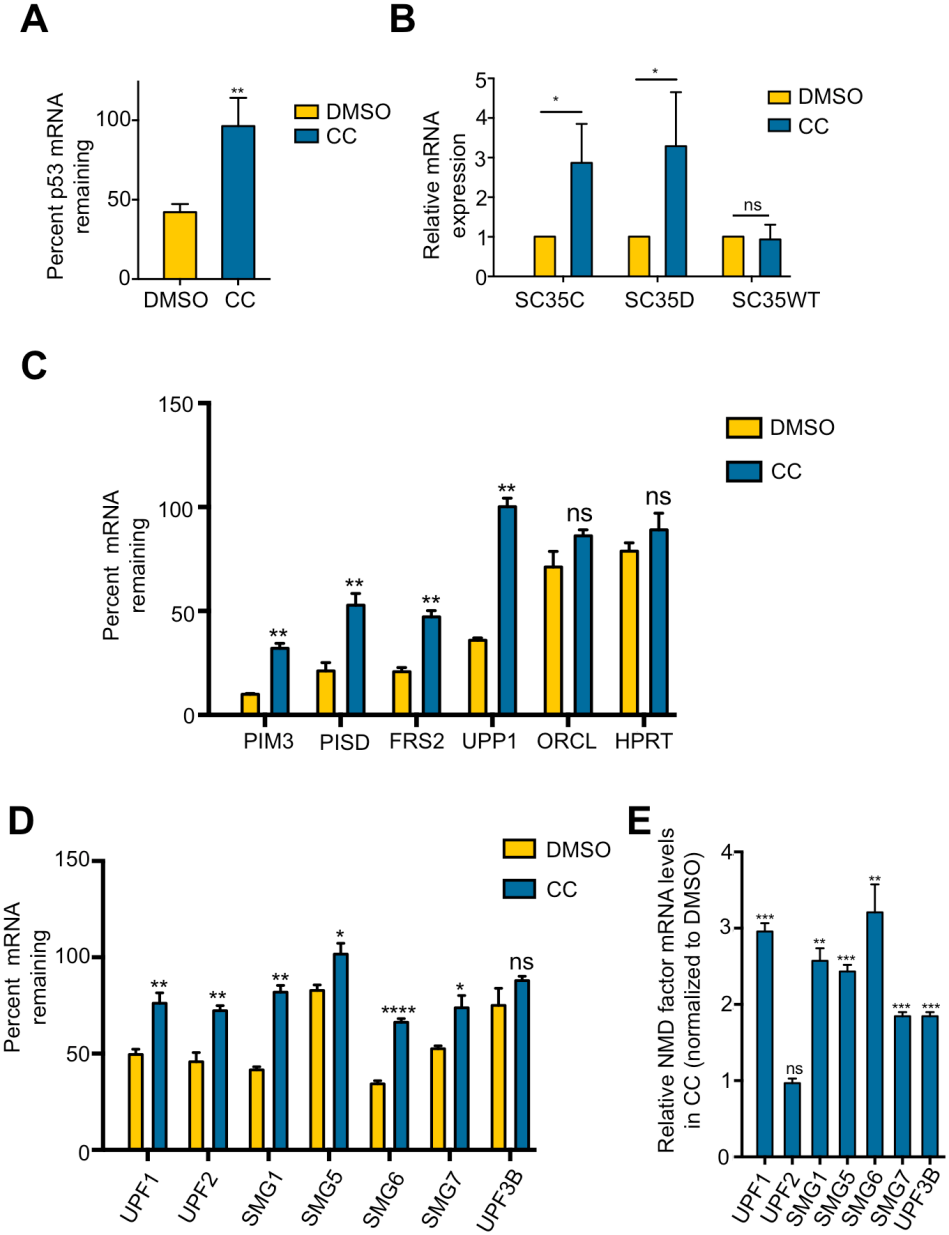
CC stabilizes endogenous NMD targets. A. Effects of CC on the stability of p53 mRNA containing a PTC in Calu-6 cells. Cells were treated with DMSO or CC (10 μM) for 24 hours followed by actinomycin D treatment for 6 hours to inhibit transcription. Total mRNA was collected before and after actinomycin D treatment. p53 mRNA levels were measured using RT-qPCR. Data represent the mean ± SD of three independent experiments. **p ≤ 0.01 (paired t-test). B. Effects of CC on the levels of the SC35 mRNA isoforms (SC35C, SC35D, and SC35WT). U2OS cells were treated with DMSO or CC (10 μM) for 24 hours followed by total RNA isolation and RT-qPCR. The mRNA levels of DMSO-treated cells were normalized to 1. Data represent the mean ± SD of three independent experiments. *p ≤ 0.05; ns, not significant (paired t-test). C. Effects of CC on the stability of known NMD targets PIM3, UPP1, FRS2, and PISD in Calu-6 cells. Samples were collected and analyzed as depicted in (A). ORCL and HPRT are non-NMD target controls. Data represent the mean ± SD of three independent experiments. **p ≤ 0.01; ns, not significant (paired t-test). D. Effects of CC on the stability of the mRNA of NMD factors UPF1, UPF2, SMG1, SMG5, SMG6, SMG7, and UPF3B in Calu-6 cells. UPF3B is not a NMD target. Samples were collected and analyzed as depicted in (A). Data represent the mean ± SD of three independent experiments. ****p ≤ 0.0001; **p ≤ 0.01; *p ≤ 0.05, ns, not significant (paired t-test). E. Effects of CC on the steady-state levels of the mRNA of NMD factors UPF1, UPF2, SMG1, SMG5, SMG6, SMG7, and UPF3B. Calu-6 cells were treated with DMSO or CC (10 μM) for 24 hours followed by total RNA isolation and RT-qPCR. Results represent fold change of mRNA levels in CC-treated cells, compared to DMSO-treated cells. ***p ≤ 0.001; **p ≤ 0.01; ns, not significant (paired t-test).

To further validate that CC is a bona fide inhibitor of NMD, we determined its effects on the stability of the endogenous mutant p53 mRNA in Calu-6 cells, which contains a PTC mutation[34]. To do this, cells were first treated with CC for 24 hrs. Subsequently, the transcription inhibitor actinomycin D was added to block new mRNA synthesis for 6 hrs. RT-qPCR was then performed to measure the levels of the p53 mutant mRNA immediately before and after actinomycin D treatment. As shown in Fig 2A, CC stabilized the PTC-containing p53 mRNA, further supporting the idea that CC inhibits NMD.

### CC stabilizes physiological NMD targets

In addition to eliminating mutant mRNAs, NMD also regulates gene expression by targeting many non-mutant physiological transcripts for degradation[35]. To further illustrate the effects of CC on NMD, we determined whether CC can also stabilize physiological NMD targets. The SC35 (SRSF2) gene encodes three splicing variants, two of which (SC35C and SC35D) are targets of NMD[36]. We found that CC treatment increased the levels of SC35C and SC35D, but had no effect on SC35WT that is not targeted by NMD (Fig 2B), consistent with the results obtained from the NMD reporter described above. In further support of the idea that CC inhibits NMD, CC also stabilized endogenous NMD targets such as PIM3, UPP1, FRS2, and PISD, but had no effect on ORCL and HPRT, which are not NMD targets (Fig 2C)[3, 37]. Previous studies have shown that transcripts of several NMD factors including SMG1, UPF1, UPF2, SMG5, SMG6, and SMG7 are themselves targets of NMD, a feature that allows for autoregulation of the NMD pathway[38, 39]. We predicted that inhibition of NMD by CC would cause stabilization of these transcripts. Indeed, CC stabilized the mRNA of these NMD factors (Fig 2D). CC treatment also increased the steady-state levels of these transcripts except UPF2 (Fig 2E). UPF3B mRNA, which is not a NMD target, was not stabilized by CC, although its steady-state level increased (Fig 2D), suggesting that CC exerted additional effects on NMD factor transcripts. Taken together, these data strongly suggest that CC has a previously unrecognized activity in inhibiting NMD in human cells.

### CC inhibits NMD independent of AMPK

**Fig 3:**
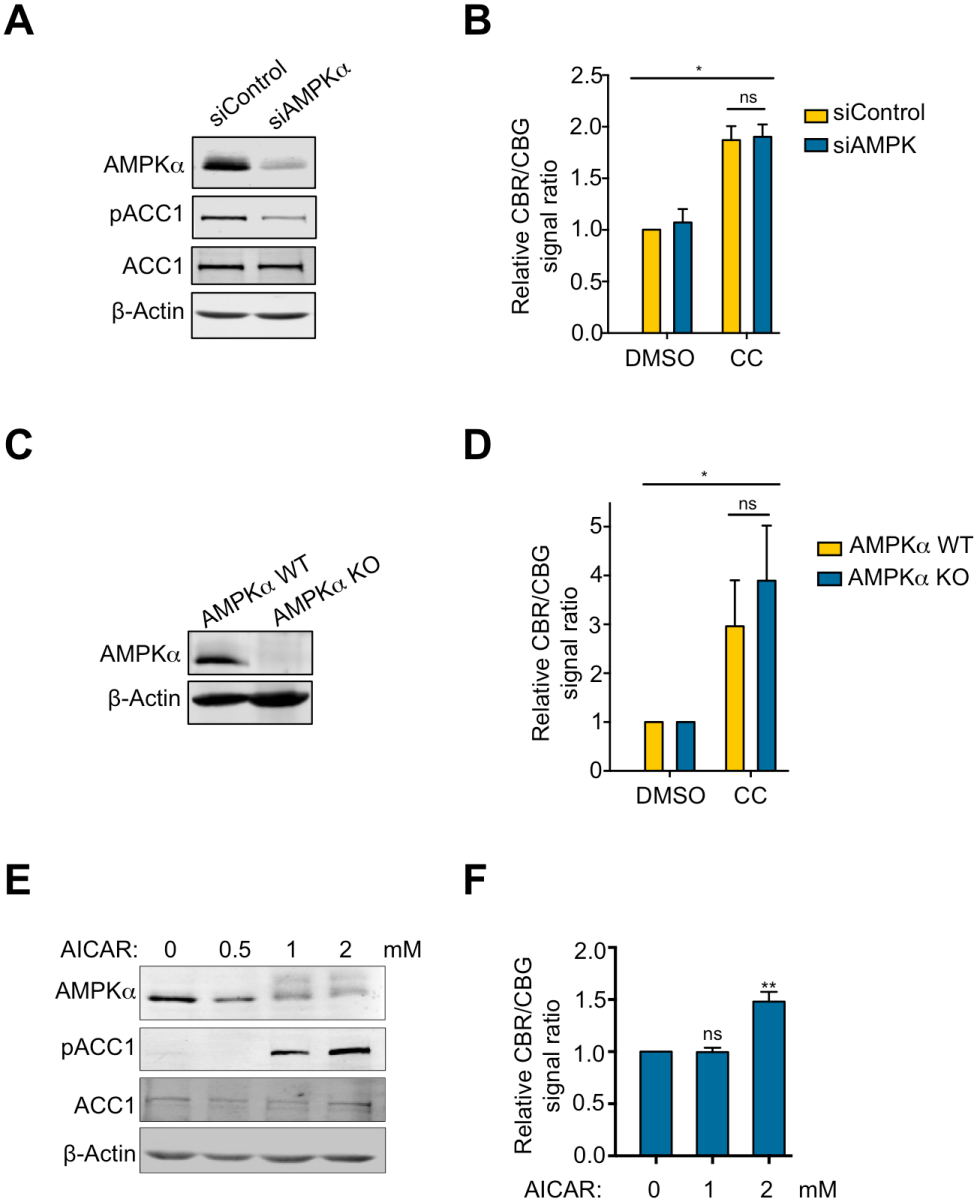
CC inhibits NMD independently of AMPK inhibition. A. Effects of AMPKα-knockdown on ACC1 phosphorylation in U2OS reporter cells. Protein samples were collected 48 hours after siRNA transfection. B. Relative ratios of CBR to CBG bioluminescence signals in AMPKα-knockdown or control-knockdown U2OS reporter cells after 24-hour treatment with 10 μM CC or DMSO. The CBR/CBG ratio of the siControl DMSO control was normalized to 1. Data represent the mean ± SD of three independent experiments. *p ≤ 0.05; ns, not significant (paired t-test). C. Knockout (KO) of AMPKα in U2OS cells. D. Relative ratios of CBR to CBG bioluminescence signals in AMPKα-KO or control cells after 24-hour treatment with 10 μM CC or DMSO and infection with adenoviruses expressing the NMD reporter. The CBR/CBG ratio of the AMPK WT DMSO control was normalized to 1. Data represent the mean ± SD of three independent experiments. *p ≤ 0.05; ns, not significant (paired t-test). E. Effects of AICAR on ACC1 phosphorylation. Activation of AMPKα by AICAR also caused a gel mobility shift. F. Relative ratios of CBR to CBG bioluminescence signals in U2OS reporter cells after treatment with indicated concentrations of AICAR. The CBR/CBG ratio of untreated control was normalized to 1. Data represent the mean ± SD of three independent experiments. **p ≤ 0.01; ns, not significant (paired t-test).

CC has been widely used as an inhibitor of AMPK, although it also targets several other kinases[23]. To determine whether CC inhibits NMD through AMPK, we used siRNAs to knock down both isoforms of the catalytic subunit of AMPK (AMPKα1 and AMPKα2) in U2OS cells. As expected, AMPK depletion reduced the phosphorylation of acetyl-CoA carboxylase1 (ACC1) at serine 79, a direct AMPK phosphorylation site (Fig 3A)[40]. However, AMPK knockdown did not influence the inhibitory effects of CC on NMD, suggesting that CC inhibits NMD independently of AMPK (Fig 3B). To rule out the possibility that the residual AMPK present in the knockdown cells was sufficient to mediate CC’s effects on NMD, we generated AMPKα-KO cells lacking both AMPKα1 and AMPKα2 using the CRISRP/Cas9 technology (Fig 3C). No significant differences in the extent of NMD inhibition were observed between WT and AMPKα-KO cells after CC treatment (Fig 3D), indicating that AMPK inhibition is not responsible for CC’s effect on NMD. Interestingly, we found that forced activation of AMPK using 5-Aminoimidazole-4-carboxamide ribonucleotide (AICAR) did not enhance, but actually attenuated NMD activity (Fig 3E and 3F). These results strongly suggest that CC inhibits NMD independently of AMPK activity and that activation, but not inhibition, of AMPK suppresses NMD.

### CC does not inhibit the kinase activity of SMG1, but it reduces the protein levels of multiple NMD factors

**Fig 4:**
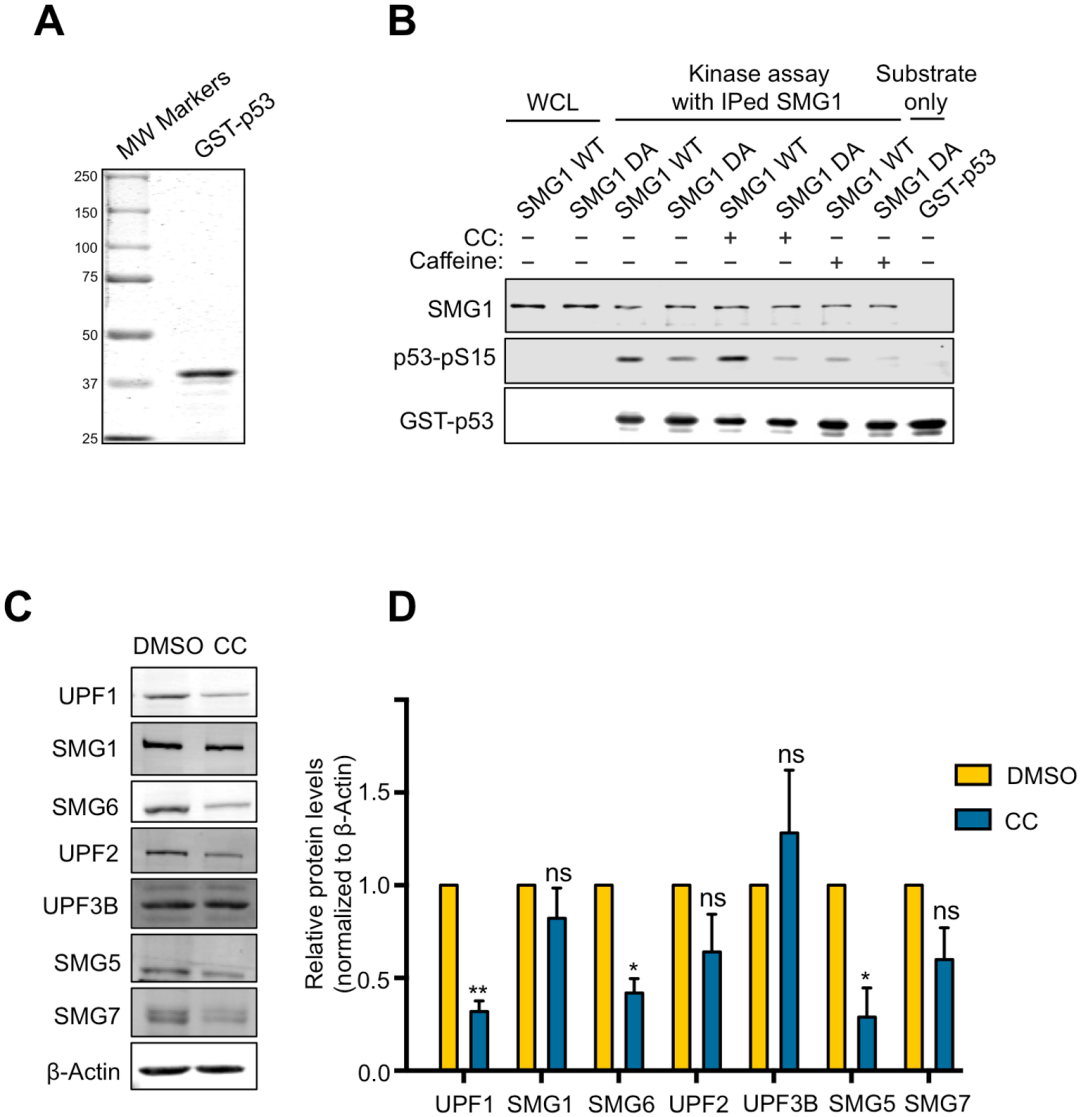
CC reduces protein levels of multiple NMD factors, but does not inhibit SMG1 kinase activity. A. Purified, recombinant GST-p53 protein containing a N-terminal fragment of p53. B. Result of *in vitro* kinase assay for SMG1 with GST-p53 as substrate containing the phosphorylation site S15. CC (10 μM) was used to test effects on SMG1 kinase activity. Caffeine (10 mM), a known inhibitor of SMG1, was used as a positive control. C. Effects of CC on the protein levels of NMD factors UPF1, UPF2, UPF3B, SMG1, SMG5, SMG6, and SMG7 in U2OS reporter cells. D. Quantification of the effects of CC on the protein levels of NMD factors UPF1, UPF2, UPF3B, SMG1, SMG5, SMG6, SMG7. Quantification was performed by measuring signal intensity relative to actin. Protein levels of DMSO-treated cells are normalized to 1. Data represent the mean ± SD of three independent experiments. **p ≤ 0.01; *p ≤ 0.05; ns, not significant (paired t-test).

As a competitive ATP analog[21], CC is known to inhibit several other kinases in addition to AMPK[23]. One possible mechanism by which CC inhibits NMD involves the inhibition of SMG1, which is the only known protein kinase that directly phosphorylates the RNA helicase UPF1 to promote NMD[34]. To test this possibility, we performed an *in vitro* kinase assay to determine whether CC can inhibit the kinase activity of SMG1. We immunoprecipitated His-tagged wild type (WT) or a kinase dead (DA) mutant of SMG1 expressed in 293T cells[34]. A purified recombinant GST-p53N fusion protein containing a N-terminal segment of p53 was used as a model substrate for SMG1 (Fig 4A)[41]. As shown before, SMG1(WT), but not SMG1(DA), efficiently phosphorylated GST-p53N (Fig 4B)[41]. This kinase activity was abolished by caffeine, a known inhibitor of SMG1[17]. However, SMG1 kinase activity was not affected by CC at 10 μM, a concentration that efficiently inhibited NMD in cells (Figs 4B and 1). This result suggests that the inhibitory effects of CC on NMD do not result from SMG1 inhibition.

We next asked whether CC affected the protein levels of NMD factors, which would impact NMD activity. Interestingly, we found that CC markedly reduced the protein levels of 3 core NMD factors including UPF1, SMG5, and SMG6 (Fig 4C and 4D). CC treatment did not affect the protein levels of SMG1, UPF2, SMG7 and UPF3B, indicating the effects of CC are specific for a subset of NMD factors (Fig 4C and 4D). This downregulation of the protein levels of multiple NMD factors may in part underlie the inhibitory effects of CC on NMD.

### CC augments the expression and stability of the NMD target ATF4, but ATF4 is dispensable for autophagy induced by CC

**Fig 5:**
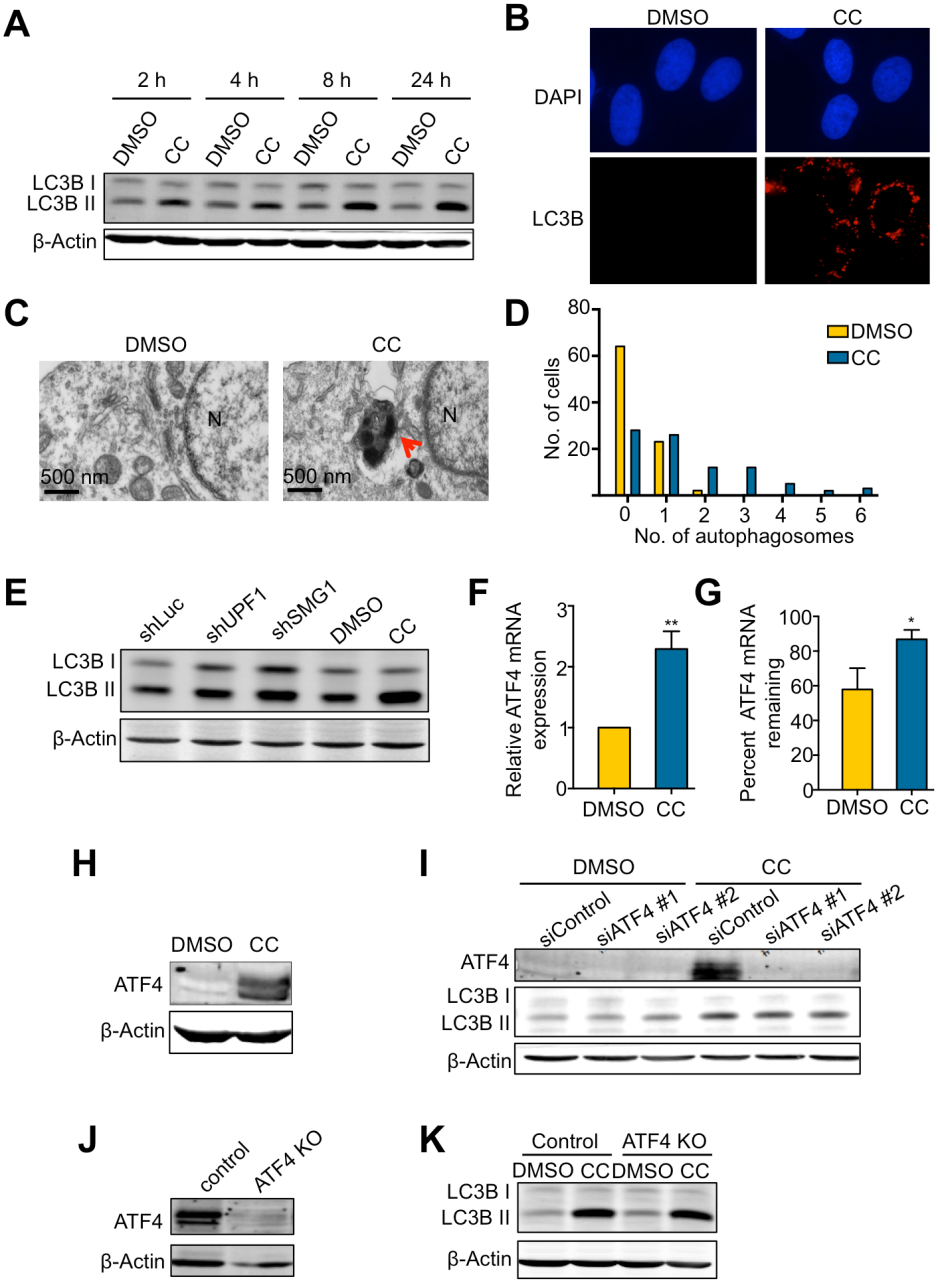
CC induces autophagy independently of the expression and stabilization of the NMD target ATF4. A. Effects of CC on LC3BII levels in U2OS cells. Cells were treated with DMSO or CC (10 μM) for 24 hours and collected at the indicated time points. B. Immunofluorescence detection of LC3B foci in U2OS cells after 24-hour treatment with DMSO or CC (10 μM). DAPI was used to visualize nuclei. C. Representative electron microscopy images of U2OS cells after 24-hour treatment of with DMSO or CC (10 μM). The arrow marks an electron-dense autophagosome. N, nucleus. D. Quantification of autophagosomes formed after 24-hour treatment with DMSO or CC (10 μM). Two sections were made from 2 different blocks for each sample. Each section is 80 nm thick and 1.75 mm long. The number of autophagosomes per cell from the two sections was counted for each sample. In total, 89 cells were counted in DMSO control, and 88 cells were counted in the CC-treated sample. E. Effects of shRNA-mediated knockdown of UPF1 or SMG1 on LC3BII levels in U2OS cells, compared to the effects in U2OS cells treated with DMSO or CC (10 μM) for 24 hours. F. Effects of CC on ATF4 mRNA expression in U2OS cells treated with DMSO or CC (10 μM) for 24 hours. mRNA expression of DMSO-treated cells was normalized to 1. Data represent the mean ± SD of three independent experiments. **p ≤ 0.01 (paired t-test). G. Effects of CC on ATF4 mRNA stability in U2OS cells. Cells were treated with DMSO or CC (10 μM) for 24 hours followed by treatment with actinomycin D for 6 hours. Total RNA was collected before and after actinomycin D treatment. ATF4 mRNA levels was measured by RT-qPCR. Data represent the mean ± SD of three independent experiments. *p ≤ 0.05 (paired t-test). H. ATF4 protein levels after 24-hour treatment with DMSO or CC (10 μM). I. Effects of siRNA-mediated knockdown of ATF4 on LC3BII levels in U2OS cells treated with DMSO or CC (10 μM) for 24 hours. J. ATF4 protein levels in WT or knockout U2OS cells. Because the basal level of ATF4 is below detection, cells were treated with 0.2 μM Thapsigargin (a known ER stressor that induces ATF4) for 4 hours before ATF4 western blot. K. Effects of ATF4 KO on LC3BII levels in WT or ATF4-KO cells treated with DMSO or CC (10 μM) for 24 hours.

Our investigation of the effects of CC on NMD was initially inspired by the observations that both CC treatment and NMD disruption induce autophagy[30, 33]. The autophagy-inducing activity of CC in cells is unexpected as AMPK, which CC inhibits, has been shown to promote autophagy[42]. To investigate the relationships between CC, NMD, and autophagy, we first determined whether CC could induce autophagy under our experimental conditions. U2OS cells were treated with 10 μM CC and the induction of autophagy was assessed. Consistent with published findings, both western blot and immunofluorescence staining results show a dramatic increase in the levels of LC3B-II, a surrogate marker of autophagosome formation, after CC treatment (Fig 5A and 5B)[43]. Using transmission electron microscopy (TEM), we also detected a marked increase in autophagosome formation after CC treatment (Fig 5C and 5D). Knockdown of the NMD factor SMG1 or UPF1 also resulted in accumulation of LC3B-II, in agreement with previous observations (Fig 5E)[33]. These data indicate that both CC and NMD disruption do indeed induce autophagy. Given the inhibitory effects of CC on NMD activity described above, these observations suggest the possibility that NMD inhibition is part of the mechanism for the autophagy-inducing activity of CC.

It has been shown that the induction of autophagy in response to NMD inhibition is in part mediated by ATF4, a direct NMD target[33]. Upon NMD inhibition, the stabilization of ATF4 mRNA leads to increased ATF4 protein levels, which promote autophagy by activating the expression of autophagy genes including LC3B and ATG5[44]. To determine whether ATF4 contributes to CC-induced autophagy, we first examined whether CC treatment causes stabilization and increase of ATF4 mRNA. RT-qPCR results indicate that CC treatment indeed increased both the stability and the steady-state levels of ATF4 mRNA (Fig 5F and 5G), leading to a dramatic increase in ATF4 protein levels (Fig 5H). To determine whether this ATF4 upregulation is important for autophagy induction after CC treatment, we knocked down ATF4 using siRNA in cells and then examined LC3B-II accumulation after CC treatment. Depletion of ATF4 expression did not significantly abrogate LC3B-II accumulation (Fig 5I). To rule out the possibility that the residual ATF4 protein in the knockdown cells is sufficient for autophagy activation, we generated ATF4-knockout cells using CRISPR/Cas9 (Fig 5J). No significant differences were observed between parental cells and ATF4-knockout cells in LC3B-II accumulation after CC treatment, further supporting the idea that ATF4 is dispensable for autophagy activated by CC (Fig 5K).

## Discussion

This study identifies CC as an inhibitor of NMD activity in human cells (Figs 1 and 2). Using both knockdown and knockout approaches, we show that the effect of CC on NMD is not mediated through the inhibition of the key metabolic regulator AMPK, adding another important “off-target” activity to CC (Fig 3A–3D)[23, 28, 45]. Interestingly, activation of AMPK by AICAR causes NMD attenuation (Fig 3E and 3F), raising the possibility that cellular metabolic state can influence NMD activity. In addition to AMPK, CC has been shown to inhibit several other protein kinases including ALK2, ALK3, ALK6, ERK8, MNK1, PHK, MELK, DYRK, HIPK2, Src, and Lck [22, 23]. SMG1 is the only protein kinase among the identified NMD factors and thus could be a potential target of CC. However, CC did not inhibit the kinase activity of SMG1 *in vitro* at the concentration that efficiently abrogates NMD in cells (Fig 4B). Interestingly, we found that CC treatment reduced the protein levels of three core NMD factors including UPF1, SMG5, and SMG6 (Fig 4C and 4D). The downregulation of these NMD factors may in part underlie the repression of NMD by CC. The mRNA levels of UPF1, SMG1, SMG5, SMG6 and SMG7, however, were higher in CC-treated cells (which was at least in part caused by the stabilization of these transcripts as a result of NMD inhibition) (Fig 2E)[38, 39, 46]. These observations raise the possibility that CC affects the mRNA translation or the stability of these NMD proteins. Several miRNAs including miR128, miR125, and miR433 have been shown to regulate NMD factors[6, 47, 48]; it will be interesting to determine in the future whether these miRNAs play a role in the CC-induced downregulation of NMD factors and attenuation of NMD efficiency.

At the cell level, both CC treatment and NMD inhibition induce autophagy (Fig 5)[30, 33]. The ability of CC to inhibit NMD suggests that NMD attenuation is part of the mechanism for the activation of autophagy by CC. Previously, it has been shown that autophagy induction after NMD inhibition is mediated in part by the stabilization of the mRNA of ATF4, a NMD target that promotes the transcription of the key autophagy factors LC3B and ATG5[33, 44]. However, we found that although CC treatment also induced and stabilized ATF4 transcripts, ATF4 was dispensable for autophagy induction by CC (Fig 5). Future work is needed to identify relevant NMD targets that promote autophagy in the presence of CC.

Our finding that CC inhibits NMD may have important implications for the use of CC as a chemical biology tool and as a potential therapeutic agent. The ability of CC to inhibit NMD may represent a mechanism for the many cellular phenotypes observed for CC. For example, in addition to autophagy, CC has been shown to induce apoptosis in many cancer cell lines and *in vivo* tumor models[28, 49–52]. Because NMD appears to be essential for human cells, its inhibition likely accounts partially for the effects of CC on cancer cell viability. CC has also been shown to induce cell differentiation, which was previously attributed in part to its inhibition of BMP type 1 receptors[53–55]. Because downregulation of NMD has been shown to promote multiple differentiation processes (e.g. embryonic stem cell differentiation, neurogenesis and myogenesis) by regulating the levels of many target mRNAs, including that involved in the BMP and TGF-β pathways in specific contexts[6, 7, 56, 57], it is possible that NMD inhibition could contribute to CC-induced cell differentiation. On the other hand, the effects of CC on many other targets also limit its use as an inhibitor of NMD in research. Thus, caution needs to taken when interpreting results of CC treatment. In spite of its many off-target activities, CC or its derivatives with low toxicity may be effective in treating certain diseases by inhibiting NMD, as NMD inhibition may alleviate the symptoms of certain genetic disorders (e.g. β-thalassemia, cystic fibrosis, Hurler’s syndrome, and Duchenne muscular dystrophy) that are caused by nonsense mutations in a single gene whose mutant protein products retain full or partial function[10]. Abrogation of NMD has also been suggested to induce antitumor immunity due to the expression of cancer antigens encoded by nonsense mRNAs[16, 58]. Thus, CC or its derivatives may have the potential of improving immunotherapy for cancers that contain an increased level of nonsense mRNAs as a result of a high mutation load. Further investigation of the mechanism of action of CC in NMD inhibition will facilitate the development of CC or its derivatives as a therapeutic agent and aid our understanding of the physiological functions of NMD.

## Materials and methods

### Key reagents

The key reagents used in this study, including plasmids, siRNAs, sgRNAs, antibodies, and chemicals are listed in Table 1.

**Table 1:** Key reagents used in the study.

Reagent	Source	Identifier
**Reporter and other plasmids**		
pBS-(CBR-TCR(PTC))-(CBG-TCR(WT))	Generated in lab	N/A
pBS-(CBR-TCR(PTC))	Generated in lab	N/A
pBS-(CBG-TCR(WT))	Generated in lab	N/A
pAdenoX-PRLS-ZsGreen1	Clontech	Cat#632258
pLenti-Cas9 Blast	Addgene	52962
pCMV-VSVG	Addgene	8454
pPAX2	Addgene	12260
plentiGuide-Puro	Addgene	52963
His-hSMG1 (WT)	Dr.Shigeo Ohno	N/A
His-hSMG1 (DA)	Dr.Shigeo Ohno	N/A
GST-p53	Dr. Robert T. Abraham	N/A
**siRNAs/gRNAs**		
siAMPK α1	Ambion	GAAGATCGGCCACTACATTC
siAMPK α2	Ambion	GAAGATCGGACACTACGTGC
siATF4#1	Ambion	S1702
siATF4#2	Ambion	S1703
siControl	Ambion	Cat#4390846
sgAMPK α1	IDT	GAAGATCGGCCACTACATTC
sgAMPK α2	IDT	GAAGATCGGACACTACGTGC
sgATF4	IDT	AACCTCTTCCCCTTTCCCCA
**Antibodies**		
Mouse anti-HA antibody	Biolegend	Cat#901507
Mouse anti-β-Actin	Thermo Fisher Scientific	Cat#MA5-15739
Rabbit anti-AMPK α	Cell Signaling Technology	Cat#2532
Rabbit anti pACC1 Ser79	Cell Signaling Technology	Cat#11818
Rabbit anti-ACC1	Cell Signaling Technology	Cat#3676
Rabbit anti-SMG1	Cell Signaling Technology	Cat#9149
Rabbit anti-p53-pS15	Cell Signaling Technology	Cat#9284
Rabbit anti-GST	Raised against GST-GFP	N/A
Rabbit anti-UPF1	Cell Signaling Technology	Cat#12040
Rabbit anti-SMG6	Abcam	Cat#AB87539
Rabbit anti-UPF2	Novus Biologicals	Cat#NB2-20813
Rabbit anti-UPF3B	Bethyl laboratories	Cat#A303-688A
Rabbit anti-SMG5	Abcam	Cat#AB129107
Rabbit anti-SMG7	Bethyl Laboratories	Cat#A302-170A
Rabbit anti-LC3B	Cell Signaling Technology	Cat#2775
Rabbit anti-ATF4	Cell Signaling Technology	Cat#11815
Alexa Fluor 488-conjugated goat anti-rabbit	Thermo Fisher Scientific	Cat#A-11008
**Chemicals**		
D-Luciferin	Sigma	Cat#L9504
Actinomycin D	Sigma	Cat#A1410
Caffeine	Sigma	Cat#C0750
Compound C (CC)	Sigma	Cat#5499
Thapsigargin	Sigma	Cat#T9033
AICAR	TOCRIS	Cat#2840

### Cell culture, transfection, lentivirus and adenovirus production and infection

Human U2OS, HEK293, HEK293T and Calu-6 cells were cultured in DMEM (Sigma, D5796) supplemented with 10% fetal bovine serum (FBS), 100 units/ml penicillin, 100 μg/ml streptomycin in a 5% CO2 incubator at 37 °C. Human foreskin BJ fibroblasts were cultured in 70% DMEM (Sigma, D5796), 15% medium 199 (Sigma, M7528), supplemented with 15% FBS, 100 units/ml penicillin and 100 μg/ml streptomycin and grown in a 5% CO2 incubator at 37 °C.

siRNA transfection for knockdown of AMPKα or ATF4 was done using TransIT-siQUEST transfection reagent (Mirus), according to manufacturer’s protocol. Transfections were done 48 hours before analysis of NMD and sample collection.

Lentiviruses expressing Cas9 or gRNAs used to knockout AMPK or ATF4 were generated by co-transfecting HEK293T cells with lentiviral vectors and packaging vectors (pCMV-VSVG and pPAX2) using TransIT-LT1 transfection reagent (Mirus). Cell culture medium containing lentiviruses was collected 48 and 72h after transfection, and used to infect target cells. Recombinant adenovirus expressing our NMD reporter was described before[19]. Target cells were infected with the reporter adenovirus for 24 hours before NMD analysis (see below).

### NMD reporter assays

A dual color bioluminescence-based NMD reporter system described previously was used to measure NMD activity in human cells[19]. The reporter in the pBluescript (KS-) vector consists of a PTC-containing TCR minigene fused to CBR (CBR-TCRβ(PTC)) and a WT TCR minigene fused to CBG (CBG-TCRβ(WT)). NMD activity is measured by the ratios of CBR-TCRβ(PTC) to CBG-TCRβ(WT) at the protein and mRNA levels using bioluminescence imaging, western blot or RT-qPCR, as previously described[19, 59]. U2OS cells stably expressing the NMD reporter system were described previously[19]. For analysis of NMD activity in Calu-6 and BJ cells, a recombinant adenovirus expressing the NMD reporter was used for infection. For bioluminescence imaging, cells were incubated with 150 μg/ml D-luciferin for 10 min at 37 °C, and bioluminescence signals were measured sequentially using IVIS 100 imager with appropriate open, red, green filters. Regions of interest (ROIs) were drawn over the images and bioluminescence signals were quantified using Living Image (Caliper) and Igor (Wavemetrics) analysis software packages as described previously[19, 60]. Spectral unmixing was performed using an Image J plugin. For RNA analysis of CBR-TCRβ(PTC) and CBG-TCRβ(WT), total RNA was extracted using a Nucleospin RNA II Kit from Clontech (740955). Reverse transcription reaction was performed to synthesize cDNA using PrimeScript RT kit from Clontech (RR037A). RT-qPCR was performed using a two-step PCR protocol (melting temperature: 95°C; annealing/extension temperature: 60°C; cycle number: 40) on an ABI V117 real-time PCR system with PowerUp SYBR Green Master Mix (Thermo-Fisher). The mRNA levels of the housekeeping genes GAPDH were used for normalization. The sequences of all the primers used are listed in Table 2. Protein levels of CBR-TCRβ(PTC) and CBG-TCRβ(WT) (both of which are N-terminally HA-tagged) were measured by western blot with anti-HA antibodies using the Li-Cor Odyssey system. Cells were lysed in 50 mM Tris, 10% glycerol, 2% SDS, 5% β-mercaptoethanol. Protein lysates were run on SDS-page gels and transferred to a PVDF membrane, blocked with casein buffer and incubated with HA antibody (Biolegend, 901507). β-Actin detected with an antibody from Thermo-Fisher (MA5-15739) was used as a loading control.

**Table 2:** Sequences of RT-qPCR primers.

Name	Sequence
CBR-F	TCCATGCTTTCGGCTTTCAT
CBR-R	CGAGAGTCTGGATAATCGCA
p53-F	GAGGTTGGCTCTGACTGTACC
p53-R	TCCGTCCCAGTAGATTACCAC
SC35C-F	GGCGTGTATTGGAGCAGATGTA
SC35D-F	CGGTGTCCTCTTAAGAAAATGATGTA
SC35C and D-R	CTGCTACACAACTGCGCCTTTT
SC35WT-F	CGTGCCTGAAACTGAAACCA
SC35WT-R	TTGCCAACTGAGGCAAAGC
UPP1-F	CCAGCCTTGTTTGGAGATGT
UPP1-R	ACATGGCATAGCGGTCAGTT
ATF4-F	ATGTCCCCCTTCGACCA
ATF4-R	CCATTTTCTCCAACATCCAATC
PIM3-F	GCACCGCGACATTAAGGAC
PIM3-R	TCCCCACACACCATATCGTAG
PISD-F	TCCCTGATGTCAGTGAACCCT
PISD-R	TGGTGTGCGTCACGAAGC
FRS2-F	TGTGGTGGAAGAGCCAGTTGT
FRS2-R	CTGAAGGCAGGCGAGCAC
ORCL-F	GGCAGCAGATGAAATCTGAA
ORCL-R	TCCAGAATGTGATTTTTGCAG
UPF1-F	CCCTCCAGAATGGTGTCACT
UPF1-R	CTTCAGCAACTTCGTGGTGA
UPF2-F	CAGGAAGAAGTTGGTACGGG
UPF2-R	ACATGCAGGGATGCAATGTA
UPF3B-F	TTTTGTTCAGGGATCGCTTT
UPF3B-R	GCTTTTTGAAAAGGTGCAAAT
SMG1-F	CTGGCAACCCAGAACTGATAG
SMG1-R	TGTAGCCACCCTTTTCGTCAT
SMG5-F	CAGTCTGAGCAGGAGAGCCT
SMG5-R	TGAAGTCGTAGCTGAGCCAT
SMG6-F	CTCTCCCATTGGAAGTACCCG
SMG6-R	CGGCGGACCAGTAGAGAAAAC
SMG7-F	CTCTGGAATCACGCCTTTAAGAA
SMG7-R	CTTCACACGGCATGGTAAATCT
GAPDH-F	CCTGTTCGACAGTCAGCCG
GAPDH-R	CGACCAAATCCGTTGACTCC
HPRT-F	TGACACTGGCAAAACAATGCA
HPRT-R	GGTCCTTTTCACCAGCAAGCT

### Analysis of the expression and stability of endogenous NMD targets

Calu-6 cells expressing a mutant PTC-containing p53 mRNA were first treated with DMSO or CC for 24 hours. Subsequently, actinomycin D (5 or 10 μg/mL) was added to inhibit new mRNA synthesis. Total mRNA was isolated both immediately before and 6 hours after the addition of actinomycin D. mRNA levels of mutant p53 was measured using RT-qPCR. The percent of mRNA remaining (stability analysis) was determined as the percent of mRNA remaining after actinomycin D treatment, compared to that before actinomycin D treatment. The stability of known physiological NMD targets including PISD, UPP1, FRS2, ATF4, PIM3, as well as transcripts of NMD factors UPF1, UPF2, SMG1, SMG5, SMG6, SMG7, and UPF3B were measured using RT-qPCR[19]. ORCL and HPRT, which are not NMD targets, were used as controls. Relative expression of different SC35 splice variants in U2OS cells was also measured using RT-qPCR. Sequences of the qPCR primers are listed in Table 2.

### Knockdown and knockout of AMPKα or ATF4 in human cells

To deplete the catalytic subunits of AMPK (AMPKα1 and AMPKα2), U2OS cells were transfected with mixed siRNAs targeting AMPKα1 and AMPKα2, respectively, using the TransIT-siQUEST transfection reagent (Mirus). To knock out AMPKα1 and AMPKα2, a Cas9-expressing U2OS cell line was first generated by lentiviral transduction with pLenti-Cas9 Blast (Addgene 52962) followed by blasticidin selection for 5 days. sgRNAs in plentiGuide-Puro (Addgene 52963) targeting AMPKα1 and AMPKα2 were then introduced into cells for gene deletion by lentivirus transduction. Cells were then plated in 96-well dishes and individual knockout cell clones were screened and verified by western blot using AMPKα antibodies. Knockdown and knockout of ATF4 in U2OS cells were also performed by the siRNA transfection and CRISPR-Cas9 methods. Because basal expression levels of ATF4 are below detection in U2OS cells, knockdown or knockout was confirmed by western blot analysis after treating cells with CC or Thapsigargin, both of which induce ATF4 upregulation[61].

### Immunoprecipitation and in vitro kinase assay

His-tagged, wild type (WT) or kinase dead (DA) SMG1 ectopically expressed 293T cells was immunoprecipitated using cobalt beads (HisPur^™^, ThermoFischer) in a buffer containing 300 mM NaCl, 50 mM Na_3_PO_4_ (pH 8.0), 0.02% Tween, 0.25% NP40, and protease inhibitor cocktail (EDTA-free, Pierce). A recombinant GST-p53N fusion protein containing a N-terminal segment of p53 with the Ser15-phosphorylation site was expressed in *E*. *coli* and purified using FPLC with a GSTTrap column (GE Healthcare). *In vitro* kinase reaction was performed by incubating His-SMG1 (WT) or His-SMG1 (DA) with the GST-p53 substrate in the presence or absence of CC (10 μM) or caffeine (10 mM) at 30°C for 2 hours in a kinase buffer (25 mM Tris HCl, pH 7.5, 10 mM β-Glycerophosphate, 0.2 mM Na_3_VO4, 10 mM MgCl_2_, 0.05 mM DTT). Reactions were terminated by the addition of SDS sample buffer. S15-phosphorylation of the GST-p53N substrate was detected by western blot using a phosphor-specific antibody (Cell Signaling Technology, 9284).

### Immunofluorescence staining to detect LC3B foci

U2OS reporter cells were plated in 3.5 cm glass-bottomed dishes (MatTek corporation) and treated with DMSO or CC for 24 hours. Cells were then washed with phosphate buffered saline (PBS, pH 7.5), and then fixed using 4% paraformaldehyde (PFA) in PBS for 10 minutes. Subsequently, cells were permeabilized using 0.2% Triton X-100 in PBS followed by blocking for 1 hour with 10% normal goat serum in PBS. Cells were then incubated overnight with anti-LC3B antibodies (Cell Signaling Technology, 2775 at 1:200 dilution in PBS with 0.1% Triton X-100). After washing, cells were incubated with Alexa Fluor 488-conjugated goat anti-rabbit secondary antibodies (Thermo-Fisher, A-11008 at 1:500 dilution) to detect LC3B foci. DNA was visualized with Hoechst 33342 staining (1 μg/ml). Fluorescence images were acquired using a Nikon Eclipse Ti-E inverted microscope with MetaMorph software, as described previously[62].

### Electron Microscopy and quantification of autophagosomes

U2OS cells were grown on 4 x 4 mm cover slips immobilized in 6 cm dishes. Cells (~70% confluent) treated with either DMSO or CC for 24 hours were briefly washed with Mammalian Ringer’s solution (155 mM NaCl, 3 mM KCl, 2 mM CaCl_2_, 1 mM MgCl_2_, 3 mM NaH_2_PO_4_, 10mM glucose, and 5 mM HEPES pH 7.2), and then fixed with 2% glutaraldehyde in buffer containing 100 mM NaCl, 2 mM CaCl_2_, 30 mM HEPES, pH 7.2 for 1 hour at room temperature. The coverslips were then washed with the same buffer 3 times over a period of 1 hour and then subjected to a secondary fixation with 1% Osmium tetroxide for 1 hour in the dark. Coverslips were washed 3 times with ultrapure water over 30 minutes and then *en bloc* stained with 1% uranyl acetate in H_2_O for 1 hour in the dark. After staining was complete, coverslips were briefly washed in ultrapure water, and dehydrated in a graded acetone series (20%, 40%, 60%, 80%, 100%) in H_2_O, with 10 minutes for each dilution. Coverslips were transferred into fresh 100% ethanol and then placed in 50% Araldite in ethanol followed by two exchanges of 100% Araldite, 30 minutes for each. Resin embedding was done by filling BEEM capsules with resin and then placing coverslips on top, with sample side facing down. To ensure infiltration of resin, blocks were incubated at RT for 2 hours, and then polymerized at 60°C overnight. Coverslips were dissolved off polymerized blocks with 42% hydrofluoric acid, followed by extensive water washes of the block before sectioning. Sections of 80 nm thickness were generated on an ultra microtome and post-stained first with 1% uranyl acetate in H_2_O for 4 minutes and then with Reynold’s lead citrate for 4 minutes. Images of sections were acquired on a JEOL 1400 Transmission Electron Microscope (JEOL, Tokyo, Japan) with an attached AMT XR111 digital camera (AMT, Woburn, MA, USA). Autophagosomes were identified as vacuoles containing cytoplasmic organelles, such as ribosomes or mitochondria, with a limited membrane as described in standard guidelines[63, 64]. Quantification was achieved by counting the number of autophagosomes per cell within two 1.75 mm sections taken from two blocks for each sample.
